# Calcium- and Phosphorus-Supplemented Diet Increases Bone Mass after Short-Term Exercise and Increases Bone Mass and Structural Strength after Long-Term Exercise in Adult Mice

**DOI:** 10.1371/journal.pone.0151995

**Published:** 2016-03-23

**Authors:** Michael A. Friedman, Alyssa M. Bailey, Matthew J. Rondon, Erin M. McNerny, Nadder D. Sahar, David H. Kohn

**Affiliations:** 1 Department of Biomedical Engineering, The University of Michigan, Ann Arbor, MI, United States of America; 2 Department of Biologic and Materials Sciences, The University of Michigan, Ann Arbor, MI, United States of America; University of Notre Dame, UNITED STATES

## Abstract

Exercise has long-lasting benefits to bone health that may help prevent fractures by increasing bone mass, bone strength, and tissue quality. Long-term exercise of 6–12 weeks in rodents increases bone mass and bone strength. However, in growing mice, a short-term exercise program of 3 weeks can limit increases in bone mass and structural strength, compared to non-exercised controls. Short-term exercise can, however, increase tissue strength, suggesting that exercise may create competition for minerals that favors initially improving tissue-level properties over structural-level properties. It was therefore hypothesized that adding calcium and phosphorus supplements to the diet may prevent decreases in bone mass and structural strength during a short-term exercise program, while leading to greater bone mass and structural strength than exercise alone after a long-term exercise program. A short-term exercise experiment was done for 3 weeks, and a long-term exercise experiment was done for 8 weeks. For each experiment, male 16-week old C57BL/6 mice were assigned to 4 weight-matched groups–exercise and non-exercise groups fed a control or mineral-supplemented diet. Exercise consisted of treadmill running at 12 m/min, 30 min/day for 7 days/week. After 3 weeks, exercised mice fed the supplemented diet had significantly increased tibial tissue mineral content (TMC) and cross-sectional area over exercised mice fed the control diet. After 8 weeks, tibial TMC, cross-sectional area, yield force, and ultimate force were greater from the combined treatments than from either exercise or supplemented diet alone. Serum markers of bone formation (PINP) and resorption (CTX) were both decreased by exercise on day 2. In exercised mice, day 2 PINP was significantly positively correlated with day 2 serum Ca, a correlation that was weaker and negative in non-exercised mice. Increasing dietary mineral consumption during an exercise program increases bone mass after 3 weeks and increases structural strength after 8 weeks, making bones best able to resist fracture.

## Introduction

Bone fragility fractures are common and costly injuries affecting more than 1.5 million people and costing $12-$18 billion in direct care each year [1]. These fractures are often attributed to reduced bone mass. Exercise has long-lasting benefits to bone health that may help prevent fragility fractures by increasing bone mass, structural-level (whole bone) strength, and tissue quality [2–6]. Bone mass (cross-sectional area, bone mineral content) and structural-level strength (yield force, ultimate force) also increase in rodents after long-term exercise programs of six-twelve weeks [7–10]. However, short-term exercise for a duration of three weeks can slow growth, limiting increases in bone mass and structural-level strength, even causing a reduction in strength compared to non-exercised controls [11,12]. Short-term exercise can still be beneficial, increasing tissue-level strength and quality (ultimate stress, damage resistance, and mineral-to-matrix ratio) [11]. The difference in bone’s response to short-term exercise at the structural and tissue levels suggests that there may be a competition for minerals needed to increase bone quantity versus bone quality, and structural- versus tissue-level strength. Bone seems to favor increasing tissue quality over increasing bone mass and structural-level strength in a short-term exercise program. The limits on bone adaptation seen in short-term exercise suggest that standard dietary amounts of mineral may be insufficient for optimal adaptation of bone in response to exercise.

Calcium (Ca) and phosphorus (P) are important regulators of bone growth and mineralization. Threshold amounts of 700–1300 mg Ca and P per day are needed from dietary sources and supplements to maintain optimal bone mass and strength [13–15]. Under healthy non-exercise conditions, dietary concentrations of Ca and P above the threshold range offer no increases in bone mass and strength [13,16]. However, during skeletal growth, there is a greater dietary recommended value for minerals than during adulthood in order to meet the demands of bone growth from modeling [14]. Exercise may similarly increase demand for minerals in order to achieve increased bone mass. Also, adding Ca supplements to a diet can restore bone mass and strength under abnormal conditions that reduce bone mass, such as following ovariectomy in rodents [17,18]. Since short-term exercise can limit increases in bone mass and structural-level strength in mice, adding mineral supplements to the diet during exercise may be a strategy to increase bone mass and structural-level strength without extending the duration of an exercise program.

Bone mass has been increased by combining exercise with a Ca-supplemented diet in human children and adolescents, but only after lengthy exercise programs [19–22]. Long-term exercise may cause a sustained increase in demand for minerals. For example, serum vitamin D_3_ increases, serum parathyroid hormone (PTH) decreases, and intestinal absorption of Ca and P increases in rodents after exercise programs of at least five weeks [10,23,24]. These changes in markers of bone metabolism with exercise suggest that even after lengthy exercise programs, there is still an increased demand for minerals, which may ultimately be what leads to increases in bone mass and structural-level strength after long-term exercise. Sustained long-term demand for minerals suggests that a standard supply of dietary minerals may not be sufficient to meet the demands from exercise.

Combining a mineral-supplemented diet with a lengthy exercise program may be able to supply sufficient amounts of minerals to prevent decreases in whole bone strength measured following short-term exercise, as well as lead to greater increases in bone mass and structural strength than longer-term exercise with a standard diet. Thus, it was hypothesized that combining a mineral-supplemented diet with exercise would prevent decreases in bone mass and structural-level strength after 3 weeks of exercise, and increase bone mass and structural-level strength after 8 weeks of exercise, compared to exercise with a standard diet.

## Methods

### Animals and Treatments–Three Weeks of Exercise x Mineral-Supplemented Diet

All animal protocols were approved by the University of Michigan University Committee on Use and Care of Animals. Eighty male C57BL/6 mice, 27.0 ± 1.4 g mean body weight, were purchased from Charles River Laboratories (Wilmington, MA) at 14 weeks of age and given 2 weeks to acclimate. Starting on experiment day 1, at 16 weeks of age, mice were randomly assigned to one of 4 weight-matched groups–a non-exercise group fed the control diet (C), a non-exercise group fed the supplemented diet (D), an exercise group fed the control diet (CE), and an exercise group fed the supplemented diet (DE). The control diet consisted of an AIN-93G diet (TestDiet®, Richmond, IN) modified by adding digestable dicalcium phosphate to contain standard concentrations of minerals– 0.5% Ca and 0.5% P. The supplemented diet was modified by adding dicalcium phosphate and calcium carbonate to contain 2% Ca and 1% P. Ca, P, and Ca:P ratio were all increased to increase serum Ca by increasing passive intestinal Ca absorption [25–27]. The control diet contained 3.90 kcal/g with an energy distribution of 65.0% carbohydrates, 16.3% fat, and 18.7% protein, while the supplemented diet had 3.69 kcal/g with an energy distribution of 63.0% carbohydrates, 17.2% fat, and 19.7% protein. All other nutrients were equivalent between the two diets. The short-term exercise program consisted of running on a 5° incline treadmill at 12 m/min, 30 min/day for 21 consecutive days [11,12]. Mice were gradually increased to a maximum speed of 12 m/min in the first 3 days of exercise. On day 22, at 19 weeks of age, mice were sacrificed, and left tibiae were harvested for analysis.

### Animals and Treatments–Eight Weeks of Exercise x Mineral-Supplemented Diet

One-hundred twelve male, C57BL/6 mice, 25.9 ± 0.8 g mean body weight were purchased from Charles River Laboratories (Wilmington, MA) at 14 weeks of age and given 2 weeks to acclimate. Starting on experiment day 1, at 16 weeks of age, mice were randomly assigned to one of 4 weight-matched groups–a non-exercise group fed the control diet (C), a non-exercise group fed the supplemented diet (D), an exercise group fed the control diet (CE), and an exercise group fed the supplemented diet (DE). The control diet was the same as used in the 3-week exercise experiment. The supplemented diet was modified to contain 5% Ca and 1% P. The control diet contained 3.90 kcal/g with an energy distribution of 65.0% carbohydrates, 16.3% fat, and 18.7% protein while the supplemented diet had 3.39 kcal/g with the same energy distribution. All other nutrients were equivalent between the two diets. A greater amount of dietary Ca was used in the 8-week experiment to attempt to increase the magnitude of effects of diet and the power for detecting effects of diet. Exercise consisted of running on a 5° incline treadmill at 12 m/min, 30 min/day for 58 consecutive days. On day 59, at 24 weeks of age, mice were sacrificed, and left tibiae were harvested for analysis.

### Cortical Geometry Measurements

Whole tibiae from 3- and 8-week groups were scanned with a voxel size of 18 μm using a GE/EVS MS-8 micro-CT specimen scanner and then analyzed with MicroView software (General Electric Healthcare, Little Chalfont, UK) and custom written Matlab (Math Works, Inc., Natick, MA) scripts. Cortical geometry metrics (tissue mineral content (TMC), volumetric tissue mineral density (vTMD), cross-sectional area, and moment of inertia about the anterior-posterior axis) were measured at a standard site and at the fracture site. TMC and vTMD were calculated using a fixed threshold and standard volume of interest. The standard site consisted of a 90-μm thick transverse section located 21.7% of the distance from the tibia-fibula junction to the proximal end of the tibia. This section is located approximately at the center of the mechanical testing region. Another 90-μm thick transverse section at the fracture site was analyzed for cortical geometry measurements used in calculations of tissue-level mechanical properties (moment of inertia about the AP axis, distance from neutral axis).

### Mechanical Testing

Structural- and tissue-level mechanical properties were measured in all 3- and 8-week experimental groups. Structural-level properties (force, deformation, stiffness, work) were measured from a 4-point bending to failure test (3-mm inner and 9-mm outer spans). Tibiae were loaded to failure with the medial side of the mid-diaphysis in tension under displacement control at 0.025 mm/sec at a data sampling rate of 30 Hz. Tissue-level mechanical properties (stress, strain, modulus, toughness) were estimated using beam bending theory with geometric measurements (moment of inertia about anterior-posterior axis, distance from centroid to medial side of the bone) from micro-CT data at the fracture site [28].

### Serum Analysis

Fasting blood samples taken before daily exercise were collected by submandibular vein bleeding. For the 3-week experiment, blood samples were collected on days 2 and 22. For the 8-week experiment, blood samples were collected 10 days before the start of the exercise program and on days 2, 30, and 59. Serum was isolated by centrifuge, and total circulating Ca and P concentrations were measured by inductively coupled plasma optical emission spectroscopy (ICP-OES). At each time point where measurements were taken, serum Ca and P concentrations from experimental groups were normalized by the concentrations of Ca and P of the control group (C) measured on the same day. ELISAs were used to measure markers of bone formation and resorption–pro-collagen type I amino-terminal peptide (PINP) and carboxy-terminal collagen crosslinks (CTX) (Immunodiagnostic Systems, Inc., Scottsdale, AZ) on samples from day 22 of the 3-week experiment and days 2 and 59 of the 8-week experiment. All manufacturers’ kit instructions were followed, including the use of the standards provided for obtaining standard curves.

### Statistical Analysis

Data from the 3- and 8-week experiments were analyzed separately. For each experiment, cortical geometry measurements, mechanical properties, and serum metabolite measurements were tested by Two-way ANOVA with Tukey’s post-hoc tests to determine if the individual effects of diet or exercise were significant (p < 0.05) and if the combined treatments had a significant interactive effect. Paired t-tests were used to compare the concentrations of serum metabolites measured at different times. Simple linear regressions were run to develop correlations between measurements of cortical bone geometry, bone metabolism markers, and bone mechanical properties. Multiple linear regressions were also run to determine the relationship between measurements of cortical bone geometry and bone metabolism markers on bone mechanical properties.

## Results

### Supplemented Diet Increased Cortical Bone Mass without Affecting Mechanical Properties after Three Weeks

**Fig 1:**
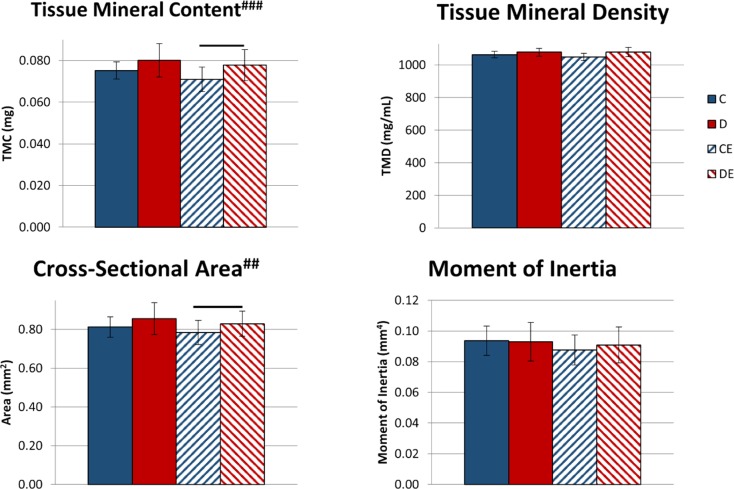
Mouse tibial cortical bone mineralization and cross-sectional geometric properties following 3 weeks of exercise x mineral-supplemented diet–tissue mineral content, volumetric tissue mineral density, cross-sectional area, and moment of inertia about the anteroposterior axis. Data shown as mean ± standard deviation. Exercised mice fed the control diet had significantly lower TMC and cross-sectional area compared to exercised mice fed the supplemented diet. ^#^Significant diet effect (##p < 0.01, ###p < 0.001, Two-way ANOVA). Groups connected by horizontal bars are significantly different (p < 0.05, Tukey’s test). C–non-exercised mice fed the control diet. D–non-exercised mice fed the supplemented diet. CE–exercised mice fed the control diet. DE–exercised mice fed the supplemented.

There were no significant differences in mean body weight between any of the groups on day 22 (28.9 ± 1.3 g for the control group, 28.7 ± 1.4 g for the supplemented diet group, 28.1 ± 1.6 g for the control exercise group, and 28.3 ± 1.6 g for the combined supplemented diet and exercise group). Exercise had no effect on tibial cortical TMC, vTMD, cross-sectional area, and moment of inertia about the anterior-posterior axis, but there was a significant effect of diet on TMC (p < 0.001, Two-way ANOVA, Fig 1) and cross-sectional area (p < 0.01, Two-way ANOVA, Fig 1). Mice on the combined diet and exercise protocol had significantly greater cortical TMC and area than mice that exercised while on the control diet (Fig 1). The supplemented diet had no significant effect on vTMD or moment of inertia.

**Fig 2:**
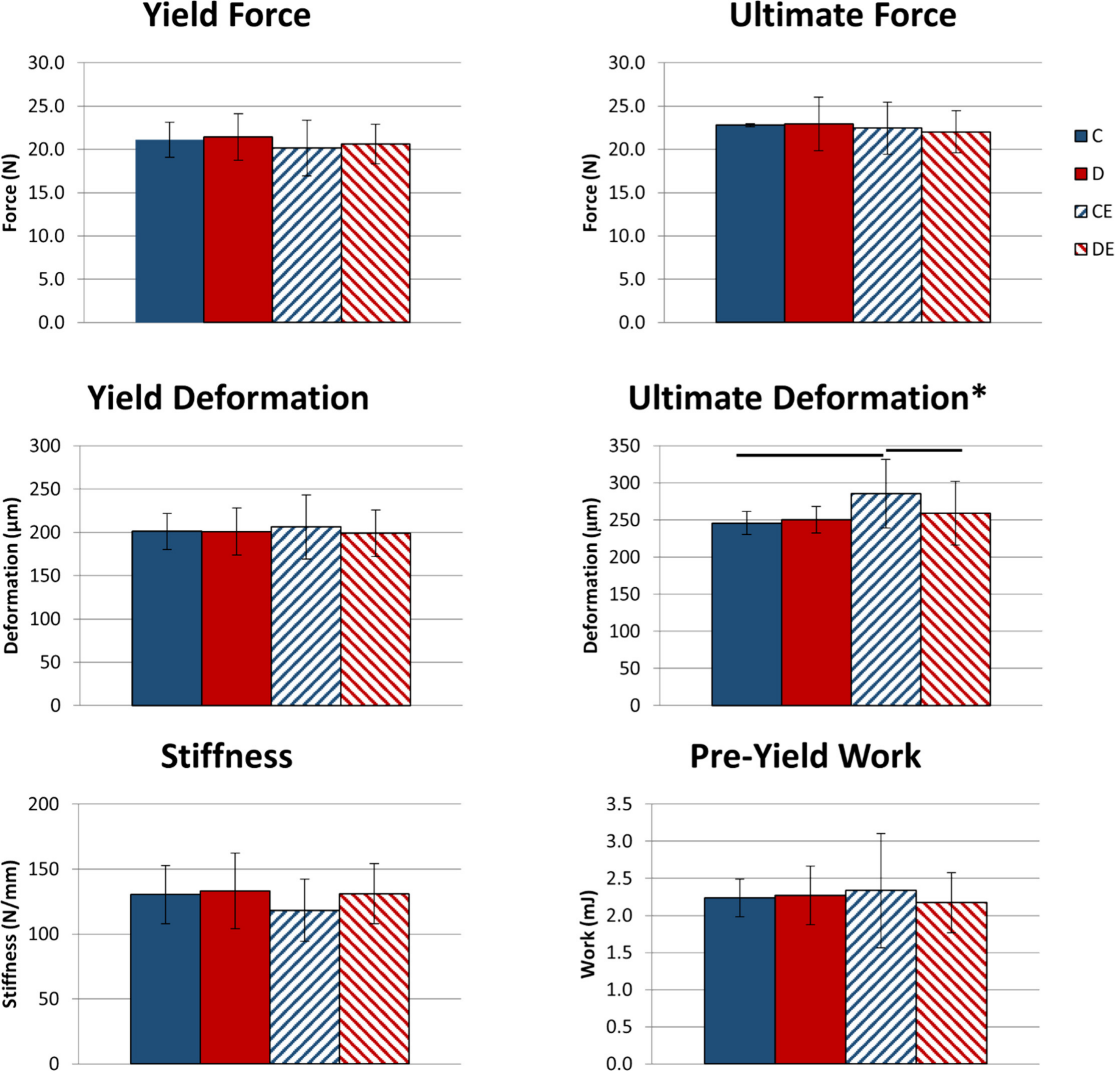
Tibial structural-level mechanical properties following 3 weeks of exercise x mineral-supplemented diet. Data shown as mean ± standard deviation. The exercised mice on the control diet had significantly greater ultimate deformation than exercised mice fed the supplemented diet and non-exercised mice on the control diet. *Significant exercise effect (p < 0.05, Two-way ANOVA). Groups connected by a horizontal bar are significantly different (p < 0.05, Tukey’s test). C–non-exercised mice fed the control diet. D–non-exercised mice fed the supplemented diet. CE–exercised mice fed the control diet. DE–exercised mice fed the supplemented.

**Fig 3:**
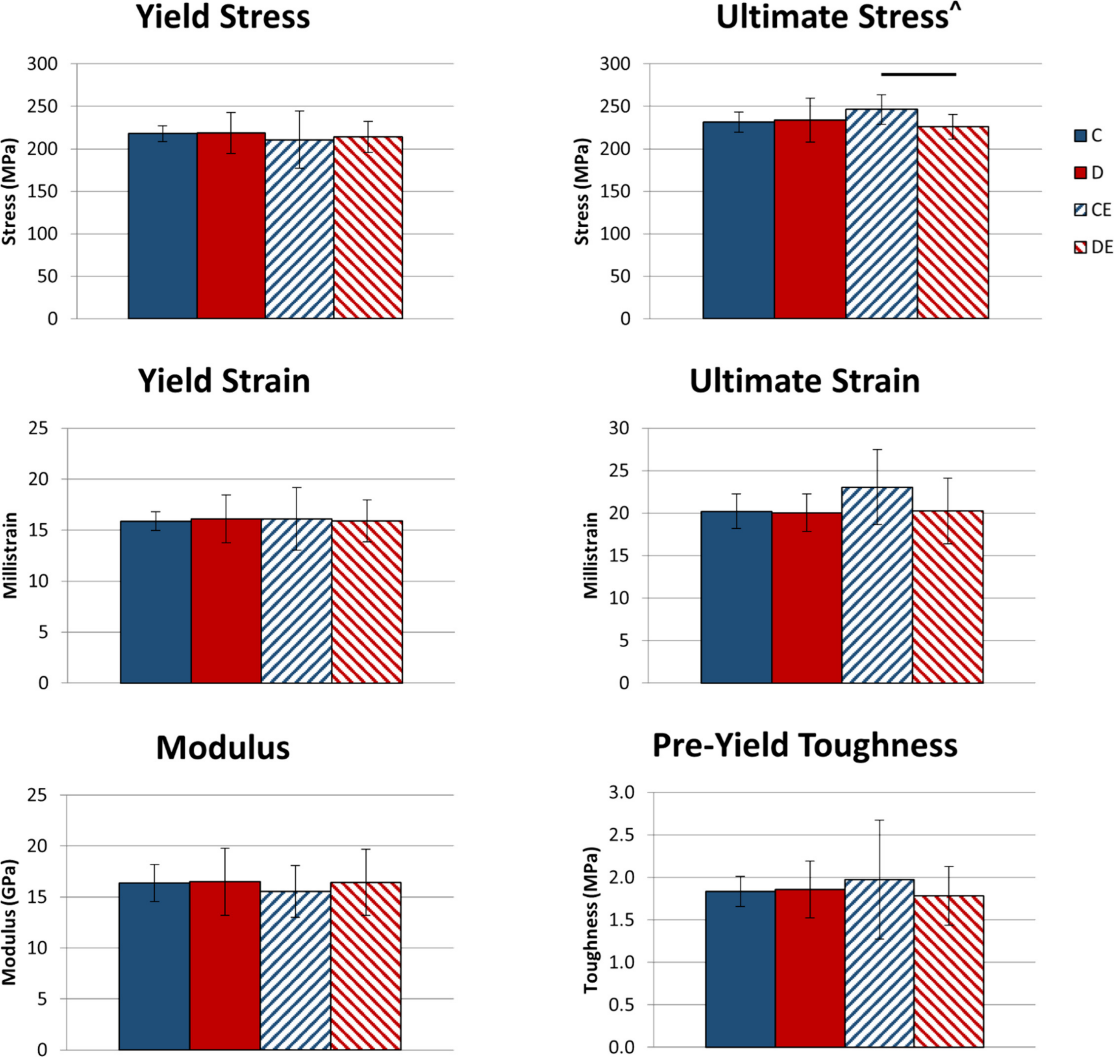
Tibial tissue-level mechanical properties following 3 weeks of exercise x mineral-supplemented diet. Data shown as mean ± standard deviation. Exercised mice on the control diet had significantly greater ultimate stress than exercised mice on the supplemented diet. ^Significant interactive effect (p < 0.05, Two-way ANOVA). Groups connected by a horizontal bar are significantly different from each other (p < 0.05, Tukey’s test). C–non-exercised mice fed the control diet. D–non-exercised mice fed the supplemented diet. CE–exercised mice fed the control diet. DE–exercised mice fed the supplemented.

For structural-level mechanical properties, there was only a significant effect of exercise on ultimate deformation (p < 0.05, Two-way ANOVA, Fig 2). Exercised mice on the control diet had significantly higher ultimate deformation than exercised mice on the supplemented diet and non-exercised mice on the control diet. No other structural-level mechanical properties were significantly affected by exercise or diet. At the tissue-level, there was a significant (p < 0.05, Two-way ANOVA, Fig 3) interactive effect between diet and exercise on ultimate stress, and the exercised mice on the control diet had significantly greater ultimate stress than exercised mice on the supplemented diet. No other tissue-level mechanical properties were significantly affected by exercise or diet, or had a significant interaction between diet and exercise.

### Exercise and Supplemented Diet Combined Increased Cortical Bone Mass and Mechanical Properties More Than Exercise or Diet Alone After Eight Weeks

**Fig 4:**
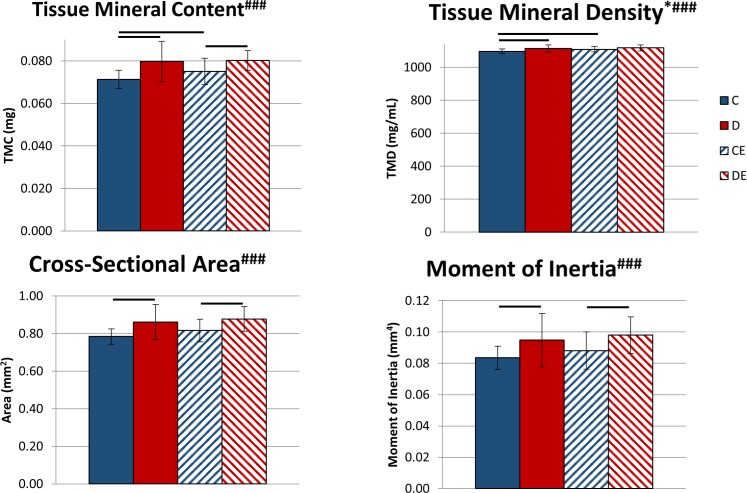
Tibial cortical bone mineralization and cross-sectional geometric properties following 8 weeks of exercise x mineral-supplemented diet. Data shown as mean ± standard deviation. The mineral-supplemented diet significantly increased tissue mineral content (TMC), cortical area and moment of inertia in exercised and non-exercised mice, and volumetric tissue mineral density (vTMD) in non-exercised mice. *Significant exercise effect (p < 0.05, Two-way ANOVA). ^#^Significant diet effect (###p < 0.001, Two-way ANOVA). Groups connected by a bar are significantly different from each other (p < 0.05, Tukey’s test). C–non-exercised mice fed the control diet. D–non-exercised mice fed the supplemented diet. CE–exercised mice fed the control diet. DE–exercised mice fed the supplemented.

Eight weeks of exercise coupled with a standard diet significantly increased TMC and vTMD (p < 0.05, Two-way ANOVA, Fig 4). The supplemented diet had a significant main effect that increased all measurements of mineralization and cortical geometry–TMC, vTMD, cross-sectional area, and moment of inertia (p < 0.001, Two-way ANOVA, Fig 4). Combining the supplemented diet and exercise led to a significant increase in tibial TMC, cross-sectional area, and moment of inertia compared to exercise alone (p < 0.05, Tukey’s tests, Fig 4). However, for mice on the supplemented diet, exercise had no effect on vTMD. Exercise did not affect cortical bone geometry for mice on the supplemented diet.

**Fig 5:**
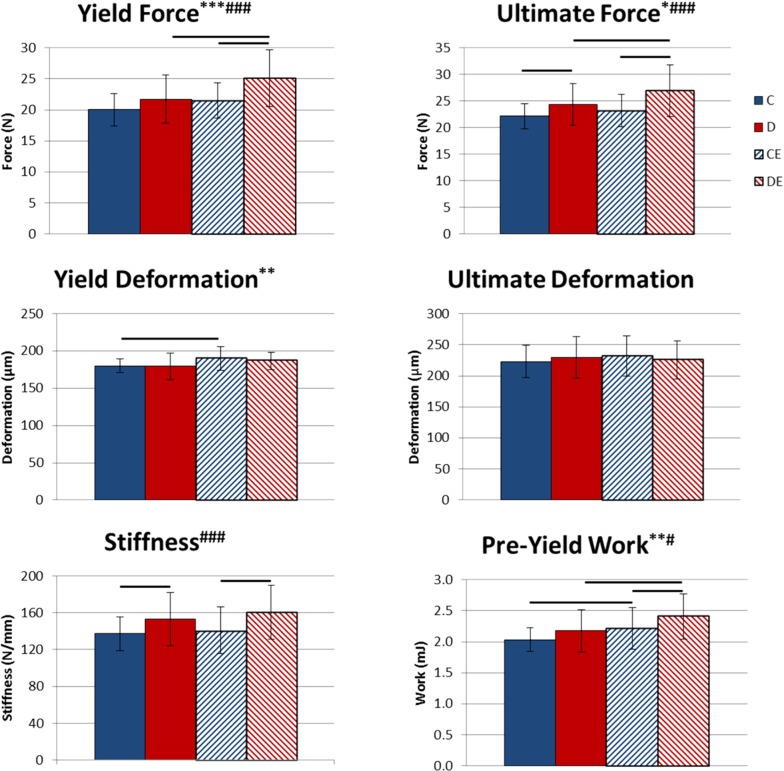
Tibial structural-level mechanical properties after 8 weeks of exercise x mineral-supplemented diet. Data shown as mean ± standard deviation. Combining the supplemented diet with exercise led to significantly greater structural strength than exercise or diet alone. *Significant exercise effect (*p < 0.05, **p < 0.01, ***p < 0.001, Two-way ANOVA). ^#^Significant diet effect (#p < 0.05, ###p < 0.001, Two-way ANOVA). Groups connected by a horizontal bar are significantly different from each other (p < 0.05, Tukey’s test). C–non-exercised mice fed the control diet. D–non-exercised mice fed the supplemented diet. CE–exercised mice fed the control diet. DE–exercised mice fed the supplemented.

**Fig 6:**
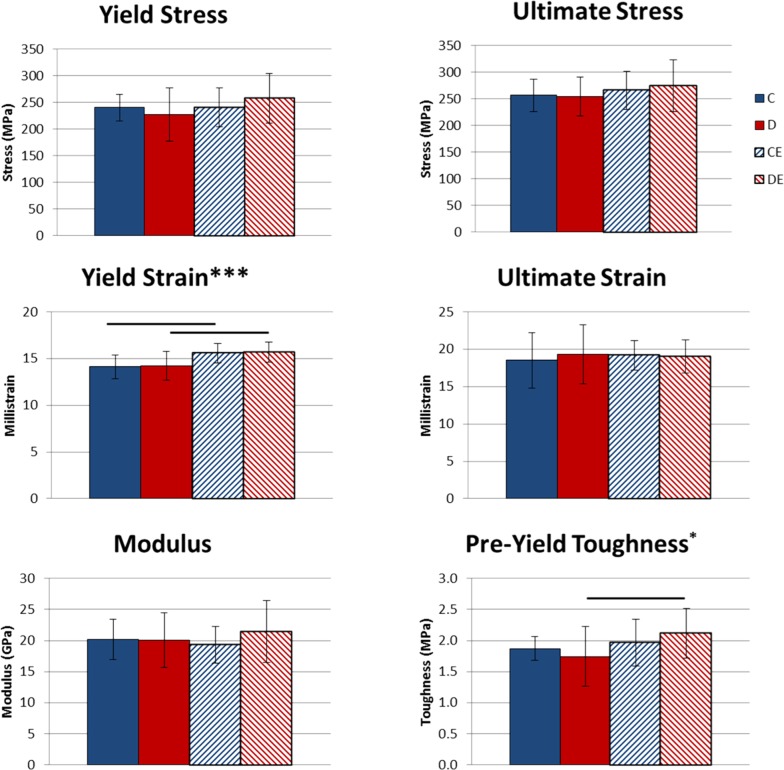
Tibial tissue-level mechanical properties following 8 weeks of exercise x mineral-supplemented diet. Data shown as mean ± standard deviation. Exercise significantly increased yield strain and pre-yield toughness. The supplemented diet did not affect tissue-level mechanical properties. *Significant exercise effect (*p < 0.05, ***p < 0.001, Two-way ANOVA). Groups connected by a horizontal bar are significantly different from each other (p < 0.05, Tukey’s test). C–non-exercised mice fed the control diet. D–non-exercised mice fed the supplemented diet. CE–exercised mice fed the control diet. DE–exercised mice fed the supplemented.

Exercise had a significant main effect that increased structural-level strength (yield force, p < 0.001 and ultimate force, p < 0.05, Two-way ANOVA, Fig 5) and deformation measures (yield deformation, p < 0.01 and pre-yield work, p < 0.01, Two-way ANOVA, Fig 5). Deformation at the tissue-level (yield strain, p < 0.001 and pre-yield toughness, p < 0.05, Two-way ANOVA, Fig 6) was also significantly increased. The supplemented diet only had significant main effects at the structural level, increasing yield force, ultimate force, stiffness, and pre-yield work (p < 0.001, p < 0.001, p < 0.001, p < 0.05, respectively, Two-way ANOVA, Fig 5). Combining the supplemented diet with exercise led to significantly greater yield force, ultimate force, stiffness and pre-yield work than exercise alone (p < 0.05 Tukey’s tests, Fig 5) and significantly greater yield force, ultimate force, pre-yield work, yield strain and pre-yield toughness than the supplemented diet alone (p < 0.05 Tukey’s tests, Figs 5 and 6). There were no differences in tissue-level mechanical properties between the exercise groups.

### Exercise Decreased Serum Bone Metabolism Markers and Increased Serum Mineral Supply as Early as Day 2 of the Program

**Fig 7:**
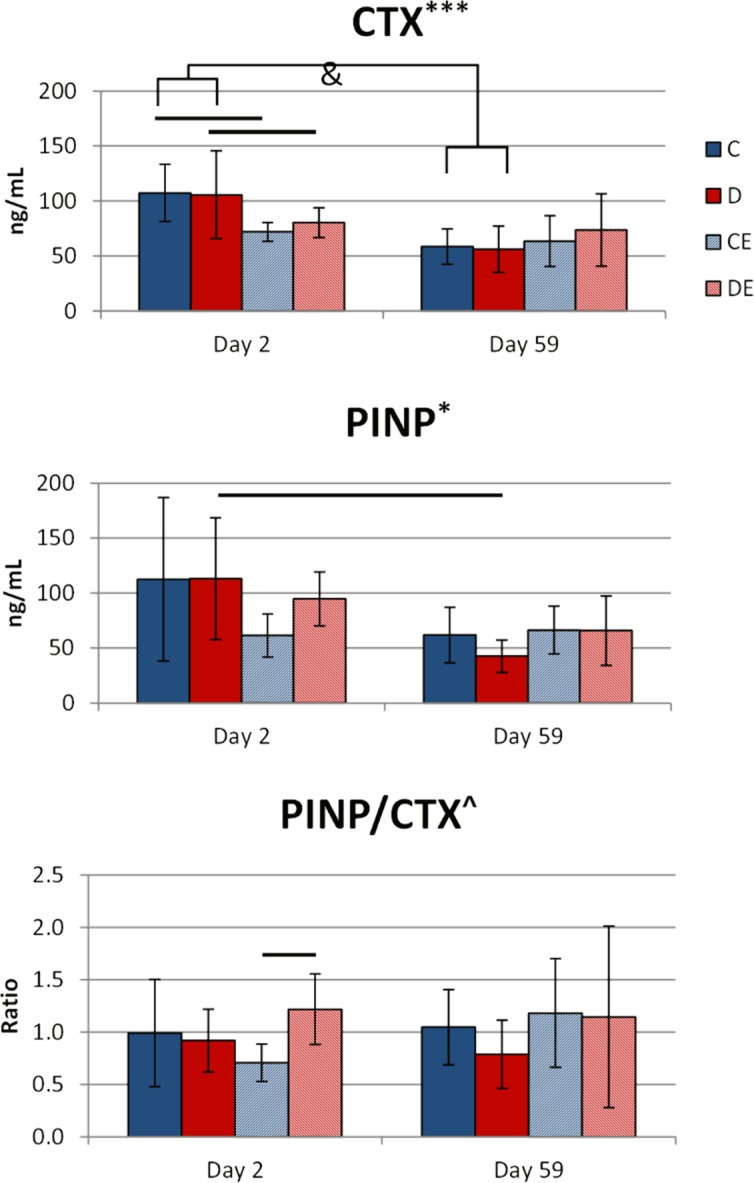
Serum CTX and PINP on Days 2 and 59 of the 8-week exercise x mineral-supplemented diet experiment. Data shown as mean ± standard deviation. CTX was significantly higher in non-exercise groups after Day 2 of the experiment (one day of treatment) and decreased to the same level as exercised mice by the end of the experiment. After 1 day of treatment, exercise with the supplemented diet caused a more formation-favored state, as indicated by the increase in PINP/CTX ratio, than exercise with the standard diet. *Significant exercise effect on day 2 (*p < 0.05, ***p < 0.001, Two-way ANOVA). ^Significant interactive effect of diet and exercise on day 2 (p < 0.05, Two-way ANOVA). &Significantly different from day 2 values (p < 0.05, paired t-test). Groups connected by a bar are significantly different (p < 0.05, Tukey’s test). C–non-exercised mice fed the control diet. D–non-exercised mice fed the supplemented diet. CE–exercised mice fed the control diet. DE–exercised mice fed the supplemented.

Exercise had a significant main effect that decreased serum CTX and PINP in mice on day 2 of the 8-week experiment (p < 0.001, p < 0.05, respectively, Two-way ANOVA, Fig 7). There were no significant main effects of exercise or diet on serum CTX, PINP, or PINP/CTX ratio in the 3-week experiment (data not shown). There were no significant group differences in CTX or PINP between the two exercise groups at any time measured in either the 3- or 8- week experiment. However, in the 8-week experiment, the combined diet and exercise group had a significantly higher day 2 PINP/CTX ratio than the exercise-only group (p < 0.05 Tukey’s test, Fig 7).

**Fig 8:**
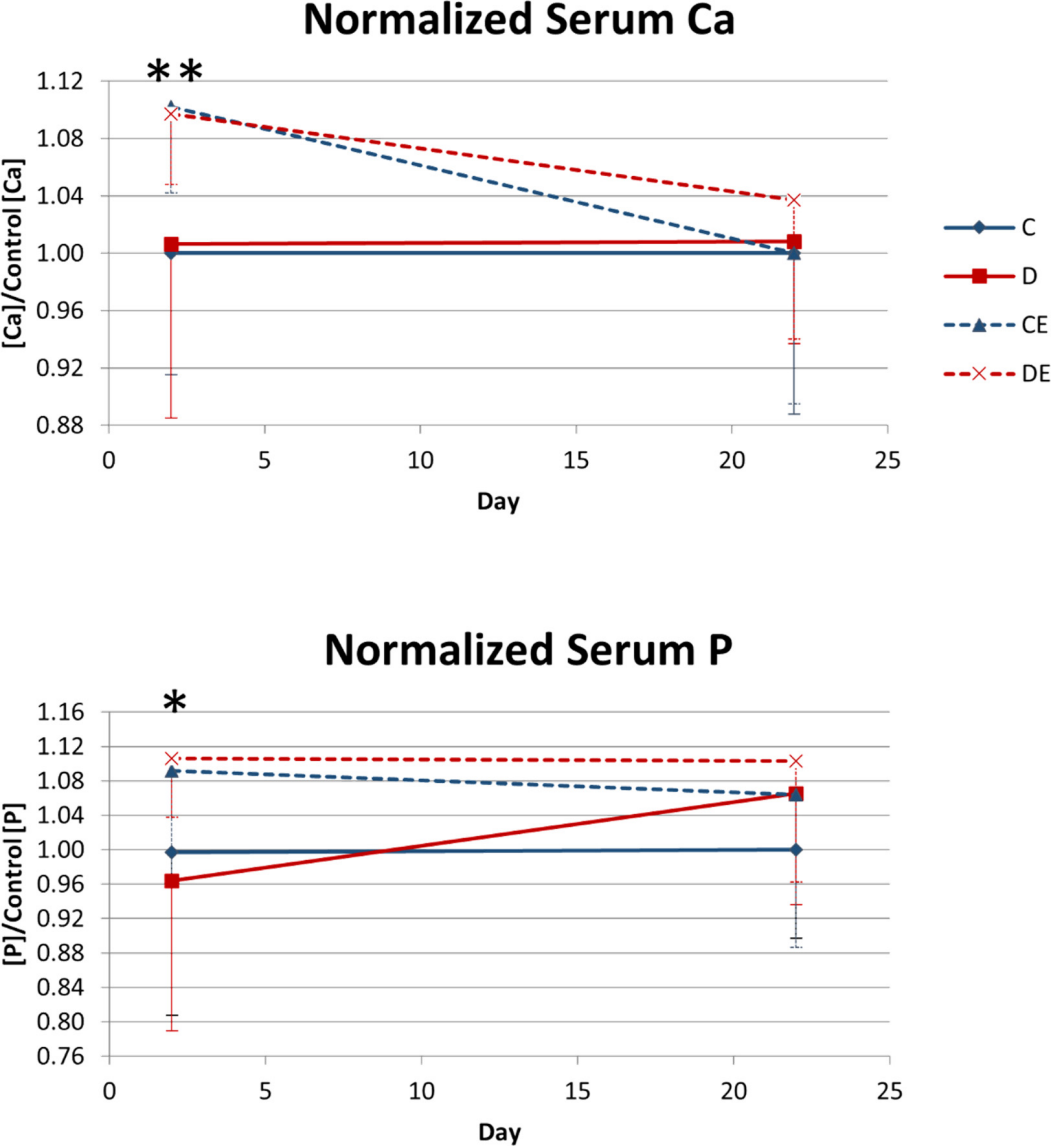
Mean serum concentrations of Ca and P on Days 2 and 22 of the 3-week exercise x mineral-supplemented diet experiment. Data are normalized to serum concentrations in non-exercised mice on the control diet at the same time point. Normalization accounts for changes with age. *Significant effect of exercise on Day 2 (*p < 0.05, **p < 0.01, Two-way ANOVA). C–non-exercised mice fed the control diet. D–non-exercised mice fed the supplemented diet. CE–exercised mice fed the control diet. DE–exercised mice fed the supplemented.

**Fig 9:**
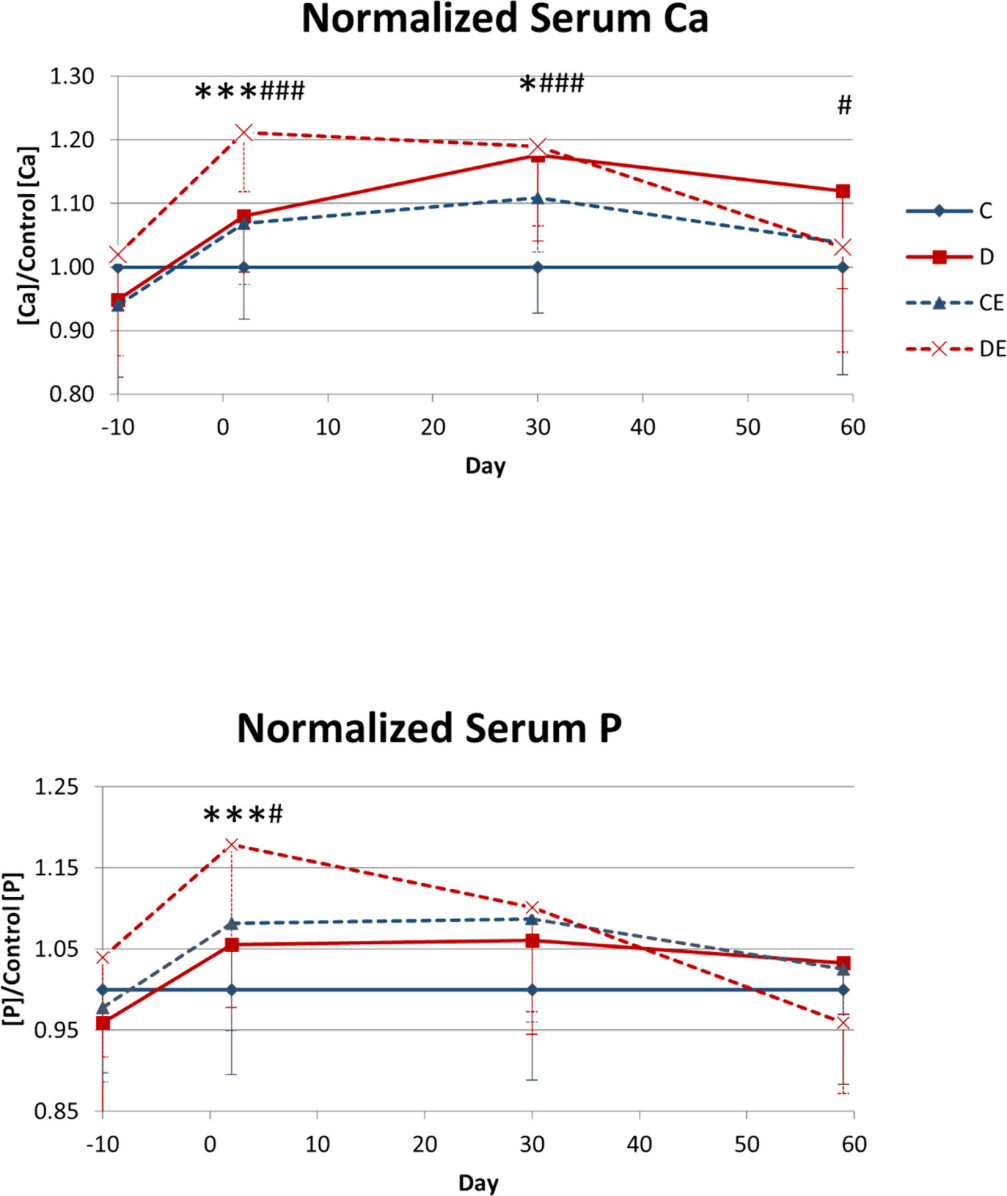
Mean serum concentrations of Ca and P 10 days before the experiment and on Days 2, 30, and 59 of the 8-week exercise x mineral-supplemented diet experiment. Data are normalized to serum concentrations in non-exercised mice on the control diet at the same time point. Normalization accounts for changes with age. After Day 2 of the experiment (one day of treatment), serum [Ca] and [P] were significantly higher than pre-experiment (0.041 < p < 0.057, 3.3 x 10^−4^ < p < 0.013 paired t-test, respectively) for the diet, control exercise and diet exercise groups, and the magnitudes of the increases were highest for mice in the diet exercise group. *Significant effect of exercise at respective times (*p < 0.05, ***p < 0.001, Two-way ANOVA). #Significant effect of diet at respective times (#p < 0.05, ###p < 0.001, Two-way ANOVA). C–non-exercised mice fed the control diet. D–non-exercised mice fed the supplemented diet. CE–exercised mice fed the control diet. DE–exercised mice fed the supplemented.

In the 3-week experiment, exercise had a significant main effect that increased day 2 serum Ca and P (p < 0.01, p < 0.05, respectively, Two-way ANOVA, Fig 8). For the 8-week experiment, exercise had a significant main effect that increased serum Ca on days 2 and 30, as well as serum P on day 2 (p < 0.001, p < 0.05, p < 0.001, respectively, Two-way ANOVA, Fig 9). The supplemented diet had a significant main effect that increased serum Ca on days 2, 30, and 59 and increased serum P on day 2 (p < 0.001, p < 0.001, p < 0.05, p < 0.05, respectively, Two-way ANOVA, Fig 9). On day 2 of the 8-week experiment, serum Ca was significantly higher in the combined supplemented diet and exercise group than in the exercise-only group (p < 0.05 Tukey’s test, Fig 9). From baseline to day 2, there was a significant increase in serum Ca in the exercise-only group (p < 0.05, paired t-test, Fig 9) and a nearly significant increase in serum Ca in the supplemented diet-only and combined diet and exercise groups (p = 0.0567, p = 0.0570, respectively, paired t-test, Fig 9). Serum P was significantly increased from baseline in these three groups (p < 0.05, paired t-test, Fig 9). Both the supplemented diet and exercise increased serum mineral supply, and combining the two increased serum Ca and P more than either treatment alone.

### Correlations Between Morphological, Biochemical and Mechanical Properties

For the 3-week experiment, cortical TMC and cross-sectional area were significantly positively correlated with yield force (r = 0.575 and r = 0.600, respectively) in non-exercised mice (Table 1). These correlations were weaker and not significant in exercised mice. In exercised mice, day 22 serum Ca was significantly negatively correlated with yield force (r = -0.379) and yield strength (r = -0.506). In the 8-week experiment, cortical TMC and area were significantly positively correlated with yield force for both exercised and non-exercised mice (0.442 < r < 0.628). In exercised mice, day 2 serum Ca was significantly positively correlated with day 2 serum PINP, r = 0.516, while for non-exercised mice there was a negative non-significant correlation of r = -0.189 (Table 2). Day 2 serum PINP was significantly positively correlated with TMC, r = 0.447, in exercised mice while non-exercised mice had a non-significant less-positive correlation of r = 0.159. The opposite effect occurred in CTX, as day 2 serum CTX was significantly positively correlated with TMC, r = 0.475, in non-exercised mice while exercised mice had a non-significant less-positive correlation of r = 0.316. Multiple linear regressions on the 3-week and 8-week experiment data did not reveal any further insight into the relations between cortical bone geometry, bone metabolism markers, and bone mechanical properties.

**Table 1:** Correlation (r) matrices for blood biomarkers, tibial cortical bone mass, and tibial mechanical properties from the 3-week experiment.

Exercised Mice									
	Day 22 PINP	Day 22 CTX	TMC	vTMD	Area	Yield Force	Yield Def.	Yield Stress	Yield Strain
Day 22 Ca	-.253	.355	.063	.142	.006	-.379*	-.310	-.506*	-.225
Day 22 PINP		.533*	.112	.360	.021	.037	-.021	.102	.048
Day 22 CTX			.229	.263	.197	.062	-.321	-.077	-.351
TMC				.644*	.943*	.141	-.123	-.365	-.015
vTMD					.401*	-.024	-.245	-.221	-.216
Area						.174	-.069	-.373	.051
Non-exercised Mice									
	Day 22 PINP	Day 22 CTX	TMC	vTMD	Area	Yield Force	Yield Def.	Yield Stress	Yield Strain
Day 22 Ca	-.050	.299	.101	.035	.067	-.063	-.392*	.025	-.192
Day 22 PINP		.235	.172	.079	.173	-.157	.101	-.082	-.054
Day 22 CTX			.344	.528*	.244	.040	-.068	.130	-.067
TMC				.531*	.974*	.575*	-.044	.210	.231
vTMD					.342*	.226	-.094	.137	.001
Area						.600*	-.008	.205	.273

TMC and area were significantly positively correlated with yield force only in non-exercised mice. In exercised mice, day 22 serum Ca was significantly negatively correlated with yield force and yield stress.

*r is significant (p < 0.05).

**Table 2:** Correlation (r) matrices for blood biomarkers, tibial cortical bone mass, and tibial mechanical properties from the 8-week experiment.

Exercised Mice									
	Day 2 PINP	Day 2 CTX	TMC	vTMD	Area	Yield Force	Yield Def.	Yield Stress	Yield Strain
Day 2 Ca	.516*	.188	.128	.169	.145	.098	-.336	.347	.067
Day 2 PINP		.373	.447*	.235	.333	.006	-.291	-.088	.093
Day 2 CTX			.316	.183	.473*	.397	-.080	.239	-.146
TMC				.435*	.972*	.589*	-.187	.040	.075
vTMD					.338*	.226	.142	.276	.184
Area						.628*	-.197	-.044	-.017
Non-exercised Mice									
	Day 2 PINP	Day 2 CTX	TMC	vTMD	Area	Yield Force	Yield Def.	Yield Stress	Yield Strain
Day 2 Ca	-.189	-.154	.013	.101	.108	-.312	.143	-.527	-.121
Day 2 PINP		.629*	.159	.067	.161	.183	-.066	-.001	-.272
Day 2 CTX			.475*	.120	.511*	.355	-.301	.180	-.160
TMC				.545*	.960*	.501*	-.351*	-.075	-.218
vTMD					.405*	.288*	-.066	.082	-.133
Area						.442*	-.365*	-.086	-.210

In exercised mice, day 2 serum Ca was significantly positively correlated with day 2 PINP, and day 2 PINP was significantly positively correlated with TMC. These correlations did not occur in the non-exercised mice. TMC and area were significantly positively correlated with yield force for both exercised and non-exercised mice.

*r is significant (p < 0.05).

Synthesizing the main effects of diet and exercise on bone cortical geometry, biochemical markers of bone metabolism and mechanical properties (Table 3) shows that exercise increased serum mineral supply and decreased serum markers of bone turnover after one day. Exercise had no impact on cortical geometry after 3 or 8 weeks, while exercise increased both structural- and tissue-level mechanical properties after 8 weeks. The supplemented diet increased serum Ca for up to 8 weeks, but did not affect bone turnover. Most measurements of mineralization and cortical geometry were increased by the supplemented diet after 3 and 8 weeks. Structural-level mechanical properties were increased with the supplemented diet only after eight weeks. Thus, exercise increased mechanical properties without affecting cortical bone mass while the supplemented diet increased cortical bone mass and structural-level mechanical properties for exercised and non-exercised mice.

**Table 3:** Significant treatment effects of exercise, diet, and diet and exercise interaction (p < 0.05 Two-way ANOVA).

	Day 2	Day 22	Day 30	Day 59
**Exercise Effects**	**↑Serum Ca, P**		**↑Serum Ca**	
	**↓Bone Turnover** (*CTX*, *PINP*)			
				**↑Cortical Bone** (v*TMD*)
		**↑Structural Properties** (*Ultimate Deformation*)		**↑Structural Properties** (*Yield Force*, *Ultimate Force*, *Yield Deformation*, *Pre-Yield Work*)
				**↑Tissue Properties** (*Yield Strain*, *Pre-Yield Toughness*)
**Diet Effects**	**↑Serum Ca, P**		**↑Serum Ca**	**↑Serum Ca**
		**↑Cortical Bone** (*TMC*, *Area*)		**↑Cortical Bone** (*TMC*, *Area*, *Moment of Inertia*, *vTMD*)
				**↑Structural Properties** (*Yield Force*, *Ultimate Force*, *Stiffness*, *Pre-Yield Work*)
**Diet x Exercise Interaction**	**Bone Turnover** (*PINP/CTX*)	**Tissue Properties** (*Ultimate Stress*)		

Exercise increased serum mineral supply and decreased serum CTX and PINP on day 2, and was associated with increases in vTMD and structural-level mechanical properties on day 59. Supplemented diet was associated with serum Ca and cortical bone geometry measurements at all time points studied, and the diet was associated with increased structural-level mechanical properties on day 59. Diet and exercise had an interactive effect on bone metabolism on day 2 and on tissue-level strength on day 22.

## Discussion

Mice exercised for 3 weeks while fed a mineral-supplemented diet with elevated Ca, P and Ca:P ratio had significantly increased cortical area and TMC, compared to exercised mice fed the control diet (Fig 1). When the protocol was extended to 8 weeks, exercised mice fed the control diet had significantly increased TMC, vTMD, and ability to deform at the structural and tissue levels compared to control mice (Figs 4–6). Combining the supplemented diet with 8 weeks of exercise significantly increased TMC, cortical area, moment of inertia, and structural-level mechanical properties compared to exercise alone (Figs 4 and 5). Increasing dietary mineral consumption during exercise therefore results in an earlier increase in cortical area and TMC that is maintained when continued for 8 weeks. These data suggest that diets containing standard amounts of Ca and P may not be sufficient for maintaining optimal bone growth throughout an exercise program.

Considering the main treatment effects of diet and exercise, the supplemented diet increased bone mass more than exercise, while exercise increased bone strength at the tissue level more than the diet. Following 8 weeks of treatment, the supplemented diet had significant positive effects on several measurements of structural-level strength and ductility, while exercise had significant positive effects on structural-level properties and several measures of tissue-level ductility (Figs 4 and 5, Table 3). After 8 weeks, the individual effects of diet and exercise were more pronounced when the supplemented diet and exercise were combined, as mice in that group had the greatest yield force, ultimate force, and pre-yield work. Thus, combined long-term diet and exercise treatments may best increase structural-level strength and ductility.

**Fig 10:**
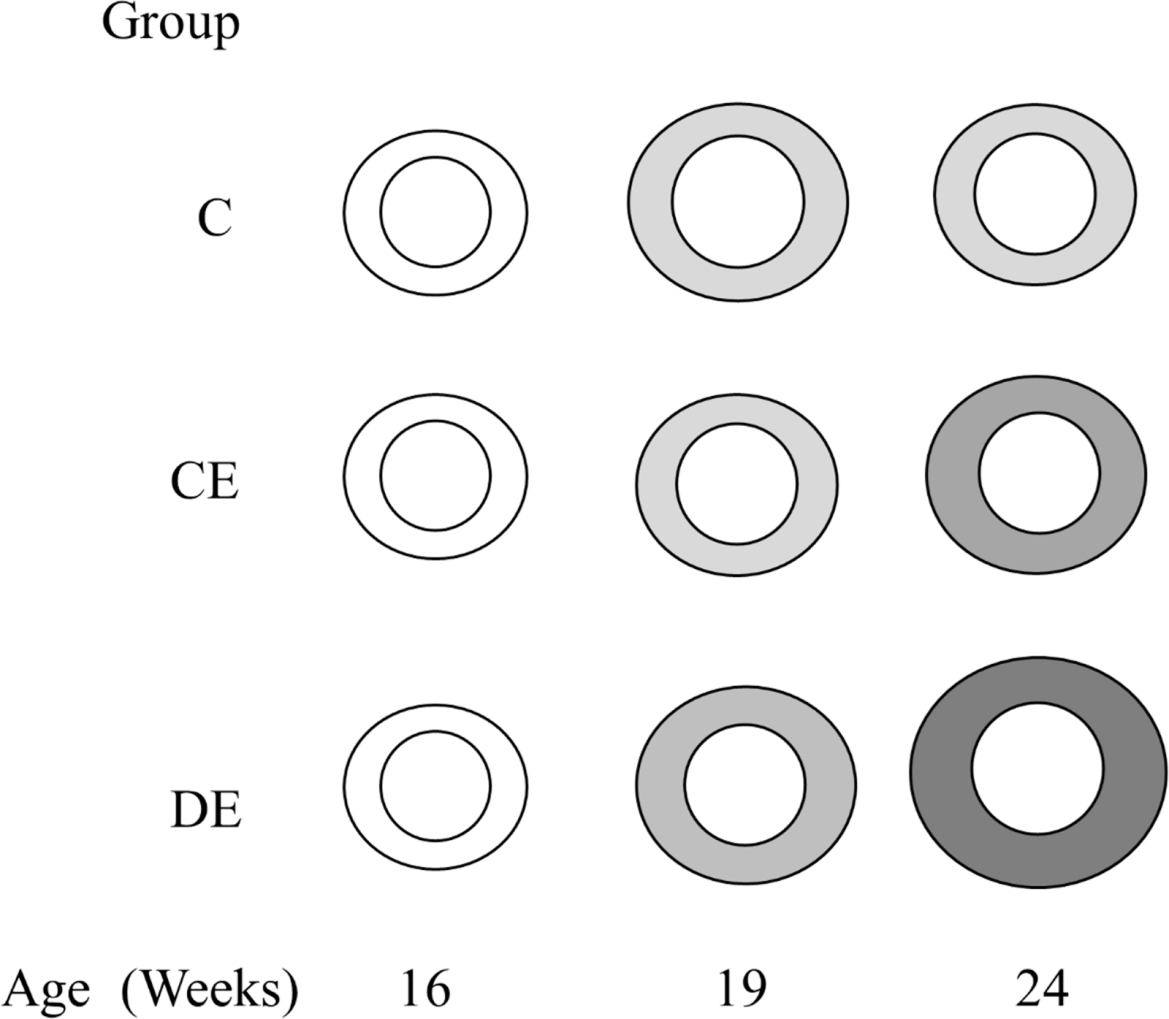
Schematic representative mouse tibial cortical cross-sectional slices from mice age 16, 19 and 24 weeks (ages of mice at 0, 3 and 8 weeks of the experiments). Darker shading denotes greater TMC. Exercise alone (CE) limits increases in area and TMC at 19 weeks, but increases area and TMC from 19 to 24 weeks. Exercise with a mineral-supplemented diet (DE) led to greater area and TMC at 19 weeks and 24 weeks compared to exercise alone.

Fig 10 shows a schematic illustration of how tibial cortical TMC and area change over time in male C57BL/6 mice subjected to mineral-supplemented diet and/or exercise intervention. Exercise alone has no effect or causes a trend towards a decrease in cortical TMC and/or area after 3 weeks [11], but exercise increases TMC and vTMD after 8 weeks. Adding the mineral-supplemented diet with exercise prevents decreases in TMC and/or area after 3 weeks, and leads to higher TMC and area than exercise alone after 8 weeks. Thus, mineral availability may be a contributing factor to short-term effects of exercise on bone mass. Increasing dietary mineral supply with exercise may be needed for maximizing cortical TMC and area for short duration exercise, and enabling adaptation at both the structural and tissue levels.

Similar to other studies, long-term exercise had a significant positive main effect on vTMD and mechanical properties at both the structural and tissue levels (Table 3) [29,30]. This 8-week exercise program increased structural-level strength, yield strain, and pre-yield toughness without increasing cortical area, suggesting changes in bone tissue quality may be occurring to increase fracture resistance independent of bone quantity. Additionally, all exercised bones had increased post-yield properties (post-yield work and deformation) compared to the non-exercised bones (data not shown). Increases in TMC and area from combining the supplemented diet and exercise did not lead to loss of post-yield strength or increased brittleness. Therefore, there may likely be tissue-level changes with exercise that increase mechanical strength independent of dietary mineral supply.

Exercise caused a quick reduction in bone turnover, as there were significant negative effects on serum CTX and PINP on day 2 of the 8-week experiment (Fig 7). Combining the supplemented diet with exercise led to a higher PINP/CTX ratio on day 2 than exercise alone. This higher formation-favored state indicates that the supplemented diet only had an effect on bone metabolism when combined with exercise. The supplemented diet did not independently affect bone metabolism at any time measured, so changes in bone formation and/or resorption that could account for the increased TMC, vTMD, area, and moment of inertia in non-exercised mice on the supplemented diet may be occurring at some intermediate time point not measured.

There were no group differences and no effects of diet or exercise on serum CTX and PINP at the end of the 8 week program (Fig 7). Thus, if the diet and exercise program had been continued beyond 8-weeks, it seems unlikely that differences in bone mass between the groups would change. There was a decline in bone turnover markers for the non-exercising mice from day 2 to day 59 (Fig 7). This change in bone turnover could be due to aging or signal a more acute shift in bone metabolism [31,32]. The mice used in this study were approximately 4 months old at the start of the diet and exercise programs, and C57BL/6 mice have reduced bone growth after 5 months of age. It is therefore possible that mice in this study were of the age where skeletal growth and development plateaued sometime during the study, causing the change in bone turnover from day 2 to day 59.

On day 2 of the 3-week experiment, exercise significantly increased serum Ca and P (Fig 8). The purpose of dietary manipulation was to increase the blood supply of minerals available for increasing bone mass. Mineral concentrations and Ca:P ratio in the supplemented diet were increased from the concentrations used in the control diet to increase serum Ca and P from passive intestinal absorption of these minerals, as was shown in Vitamin D receptor knockout mice [26]. In Vitamin D receptor knockout mice, increasing dietary Ca alone, Ca and P, or Ca:P ratio all individually increased serum Ca and P. We hypothesized that increasing Ca:P ratio and Ca and P concentrations would cause an even greater increase in absorption of these minerals than increasing ratio or mineral concentrations alone.

The 2% Ca-supplemented diet alone did not have a significant effect on serum Ca or P. Therefore, dietary Ca was elevated to 5% in the 8-week experiment to increase serum mineral supply and the effects of the mineral-supplemented diet. On day 2 of the 8-week experiment, both the supplemented diet and exercise significantly increased serum Ca and P, and the concentrations of Ca and P were further increased when the mineral-supplemented diet and exercise were combined (Fig 9). The increase in serum Ca and P with exercise indicates that exercise increased serum mineral availability independent of dietary supply. There could be separate mechanisms for increasing serum Ca and P with the supplemented diet or exercise, and they may work together to cause greater serum mineral concentrations when the supplemented diet and exercise are combined [14,33].

Serum P did not remain elevated with diet or exercise beyond day 2 (Fig 9). Dietary P was increased in the supplemented diet to increase the supply of serum P for increasing bone mass. Since increasing dietary Ca alone has little effect on serum Ca and bone mass [16,34,35], dietary P was increased in an attempt to increase passive intestinal absorption of Ca and total serum mineral supply. Additionally, increasing dietary P can increase TMC and bone structural strength when dietary Ca is also increased [25,26]. The amount of P in the supplemented diet was lower than Ca, which may explain why serum P was not elevated for the same duration or magnitude as Ca. The dietary P required for increasing bone mass may not be as high as Ca, so exercise may not cause as high an increase in demand for P as for Ca. To our knowledge, this is the first study to show exercise can have an effect on bone turnover and serum mineral levels within 24 hours in mice. With such early rapid changes in bone metabolism and mineral demand, increasing dietary mineral supply of both Ca and P at the start of exercise programs may be necessary to increase PINP/CTX ratio to maximize gains in bone mass with exercise.

After 3 weeks, cortical TMC and cross-sectional area were significantly positively correlated with yield force in non-exercised mice (Table 1). These correlations were weaker and non-significant in exercised mice. This difference suggests increasing bone mass may not be as beneficial towards increasing structural strength with short-term exercise. After 8 weeks of exercise, cortical TMC and area were significantly positively correlated with yield force in both exercised and non-exercised mice (Table 2). Thus, for lengthier exercise programs, increases in bone mass could be more beneficial towards increasing structural strength.

There was a different relation between serum Ca and PINP, depending on whether or not the mice were exercised (Table 2). In the mice exercised for 8 weeks, there was a significant positive correlation between day 2 serum Ca and day 2 serum PINP (r = 0.516, Table 2). PINP was also a stronger predictor of cortical bone mass measurements (TMC, vTMD, and area) in exercised mice than non-exercised mice. Increasing PINP early in an exercise program by increasing serum Ca may be most beneficial for increasing bone mass long-term in exercised mice. In non-exercised mice, day 2 serum Ca was negatively, but non-significantly correlated with PINP and CTX, suggesting increasing blood Ca supply has minimal effect on or may suppress bone turnover without exercise. Thus, increasing dietary Ca consumption is more impactful to bone turnover when combined with exercise.

Dietary mineral supply may affect the timing of exercise effects on bone. With the lower supply of minerals in the control diet, it is possible that exercised mice prioritized increasing tissue-level mechanical properties over increasing cortical TMC and area after 3 weeks, as ultimate stress was higher in the exercise-only group than in the combined supplemented diet and exercise group (Fig 3). However, exercised mice on the supplemented diet may have prioritized increasing cortical area over tissue-level mechanical properties after 3 weeks. Differences in tissue-level mechanical properties between the exercise groups were not present after 8 weeks, suggesting mineral availability may only affect short-term changes to tissue-level properties.

Bone size, shape and architecture (cortical area, thickness, and section modulus) are contributing factors for preventing fractures [36]. Because fractures are a failure of bone to adapt to loading, increasing bone size, strength, and damage resistance may help prevent fractures. Short-term exercise can increase fatigue damage resistance in mice [11]. Exercise also increases mineral-to-matrix ratio and decreases carbonate/phosphate ratio, possibly because exercise favors increasing tissue quality over increasing bone mass. If minerals are in short supply, increasing mineralization of existing tissue could be favorable to adding new tissue and increasing bone mass. This could increase tissue-level properties in a shorter time span. However, by not increasing bone mass in a short-term exercise program, bone would still be subjected to repetitive overloading that can lead to fractures. Since the mineral-supplemented diet increased bone mass in exercising mice after 3 and 8 weeks of exercise, dietary intervention also may be useful in preventing exercise-related fractures. Further work needs to be done to examine the effects of combining the mineral-supplemented diet with exercise on bone’s resistance to fatigue damage and other tissue-level properties.

Most studies that examine dietary mineral requirements for optimal bone growth and bone mass accumulation do not involve exercise or only compare standard dietary Ca to diets insufficient in Ca [30,34]. As exercise creates an increased demand for minerals and alters bone metabolism, the threshold amounts of dietary minerals needed for optimal bone growth may be higher with exercise. Increasing dietary mineral consumption during an exercise program could increase blood mineral supply and allow greater increases in cortical TMC and area in the short term, as well as increasing structural-level strength long term.

## Supporting Information

S1 Table 3-Week and 8-Week Experiment Measurements. (XLSX)
